# Still Another New Hypnotic

**Published:** 1890-04

**Authors:** 


STILL ANOTHER NEW HYPNOTIC IS "Somnol." It is an ethyl-chloral-urethane (7 $H_{12}Cl_3O_3N$). It is given in doses of about thirty grains. It has been discovered by M. Radlauer, of Berlin.—*Medical Record.*

